# The Third Man: hierarchy formation in Wikipedia

**DOI:** 10.1007/s41109-017-0043-2

**Published:** 2017-07-27

**Authors:** Jürgen Lerner, Alessandro Lomi

**Affiliations:** 1 Department of Computer and Information Science, University of Konstanz, Universitätsstr. 10, Konstanz, 78464 Germany; 2 Faculty of Economics, Università della Svizzera italiana, Via Buffi 13, Lugano, 6904 Switzerland; 3 University of Southern California, Los Angeles, 90007 United States

**Keywords:** Hierarchy formation, Online collaboration networks, Open production, Relational event models, Wikipedia

## Abstract

Wikipedia articles are written by teams of independent volunteers in the absence of formal hierarchical organizational structures. How is coordination achieved under such conditions of extreme decentralization? Building on studies on the organization of dominance relations in animal and human societies, we theorize that coordination in Wikipedia is made possible by an emergent hierarchical order sustained by self-organizing sequences of text editing events. We propose a new method to turn the editing history of Wikipedia pages into an evolving multiplex network resulting from three types of interaction events: dyadic undo, dyadic redo, and third-party based edit events. We develop new relational event models for signed networks that specify how the probability of observing various types of edit events depends on their embeddedness in sequences of past edit events. Using a random sample of page histories comprising 12,719 revisions produced by 7,657 unique users, we examine the relation between theoretically defined sequences of text editing events, and the emergence of linear dominance hierarchies that regulate production relations within Wikipedia. We find evidence that dyadic interaction gives rise to systematic extra-dyadic dependence structures that are partially consistent with a hierarchical interpretation of the Wikipedia editing network. We support and complement the statistical analysis of multiplex event networks with data visualizations that provide qualitative validation of our main results.

## Introduction

The formation of dominance hierarchies taking the classic “pecking order” structure is commonly observed across a variety of taxa (Chase 1980; Jameson et al. 1999). Available evidence in support of this claim typically comes from studies of interaction within small groups observed in experimental settings (Chase 1982), and, occasionally, from studies based on simulation experiments (Dugatkin and Earley 2003; Beacham 2003).

Empirical studies on hierarchy formation in larger and open human groups are still relatively infrequent (see Fişek et al. 1991 and references therein). In this paper we focus on Wikipedia as an opportunity to analyze large-scale, longitudinal, and directly observable data on endogenous hierarchy formation in a naturalistic (non-experimental) setting. The almost complete absence of exogenous elements of formal organization makes Wikipedia uniquely useful to study how hierarchical order may emerge from sequences of social interaction events.

Text editing activities in Wikipedia also provide a rare occasion to study social behavior where individual “participation to collective action” (adding text to an article (Zhang and Zhu 2011)), “acts of aggression” (deleting text that others have added (Sumi et al. 2011)), and “expressions of solidarity” (re-instating deleted text (Brandes et al. 2009a)) may be *directly* observed in large numbers over an extended period of time. How participation in collective action, aggression, and solidarity produce and reproduce social order is one of the most enduring questions posed by economic sociology (Elster 1989) – and by sociology more generally (Doreian and Fararo 2012; Durkheim 2014).

In this paper, we adopt and extend core analytical concepts from behavioral ecology research on the social dynamics of hierarchy formation in animal societies (Chase 1980; Chase et al. 2002; Dugatkin 1997) to examine how sequences of local interaction events observed in the course of editing activities cumulate into dominance hierarchies that impose a global order on the production relations of Wikipedia pages. We focus on symmetry and transitivity in co-editing relations — the core conditions for the emergence of linear dominance hierarchies as partially ordered sets of individuals (Dugatkin and Earley 2003).

Our analytical objective in this paper is to establish the extent to which the sequential development of dominance relations that are actually observed (Chase 1982) give rise to a global hierarchical ordering that cannot be directly observed, but that shapes future sequences of interaction among individuals. This objective is both theoretically important as well as empirically relevant. It is theoretically important because we clearly would like to know more about the conditions under which social structure emerges from decentralized interaction among autonomous social agents (Gould 2002). It is empirically valuable because we still have an imperfect understanding on how voluntary provision of public goods with economic value – like Wikipedia articles – can be coordinated in the absence of formal hierarchies and incentive systems (von Hippel and von Krogh 2003; Lerner and Tirole 2001).

More specifically, our work makes two main contributions to social network research on hierarchy formation. First, using newly developed statistical models for relational events, and building on recent work from Butts (2008) and Brandes et al. (2009b), we test the hypothesis that sequences of decentralized editing events sustain linear hierarchical ordering in open productions such as Wikipedia. The relational event models (REM) that we implement in this paper considers individual editing events as the elementary components of an evolving multiplex network, encoding the complete history of past interaction among users. Unlike many existing studies, we are not interested in deriving aggregate measures of hierarchy, but in identifying the relational micro-mechanisms through which individual agents *construct* macro-level order.

Second we complement and validate the results of statistical analysis with network visualizations designed and implemented to represent the global hierarchical structure of Wikipedia edit networks. The network visualizations show the distribution of status in the network and help to identify violations of linear hierarchical ordering among Wikipedians involved in editing activities. We go beyond existing studies by showing that third-party (“bystander”) effects are more reliable and robust predictors of status hierarchies than patterns of dyadic interaction.

In this article we build on – and extend – the prior work of Lerner and Lomi (2017) to examine patterns of text undo, redo, and third-party edit events in a random sample of Wikipedia articles comprising 12,719 revisions by 7657 unique users. The results of the analysis reveal that third-party (“bystander”) effects operate over and above “pure winner” and “pure loser” effects (Chase et al. 1994; Dugatkin 1997), i. e., over and above the tendency of dominant (or dominated) participants in a dyad to continue dominance (or continue to be dominated). This result is important because it reveals the presence of extra-dyadic self-organizing tendencies in the production of articles consistent with the presence of linear hierarchies in Wikipedia.

## Theoretical background and hypotheses

### Linear dominance hierarchies

If for all pairs of members (dyads) in a society, individuals are either dominant or submissive (but not both), and if all existing relations among sub-groups of three individuals (triads) are transitive, then that society as a whole will be organized according to a linear dominance hierarchy – also known as “pecking order” (Chase 1982; Shizuka and McDonald 2015).

The presence of linear dominance hierarchies is commonly observed in animal societies, (e. g., Schjelderup-Ebbe 1922; Chase 1982; Boyce et al. 2012). Despite obvious differences in individual attributes and behavior, a similar hierarchical organization of dominance relations has been frequently observed also in small human groups (Chase 1980; Skvoretz and Fararo 1996). In both animal, as well as human societies the emergence of hierarchical order may be interpreted as an outcome of sequential interaction between individuals (Chase 1982), rather than as a consequence of individual differences (Chase et al. 2002; Chase and Lindquist 2016). Extant research emphasizes the role of constructive mechanisms of hierarchy formation based on sequential interaction, rather than aggregate measures of hierarchization defined at the system level (Mones et al. 2012; Czégel and Palla 2015; Corominas-Murtra et al. 2013). More specifically, the narrow focus on anti-symmetry and transitivity in dominance relations is justified by the fact that these (local) properties of social relations imply the presence of a linear dominance hierarchy.

We innovate by developing a dynamic network approach to the emergence of linear hierarchies from sequences of social interaction events (Shizuka and McDonald 2012; 2015). Building directly on recent work on dynamic network motifs in animal hierarchies (Chase and Lindquist 2016), we specify new relational event models event models (Butts 2008; Brandes et al. 2009b; Stadtfeld and Geyer-Schulz 2011; Vu et al. 2017) that allow us to examine the consistency of mechanisms of *change* in local configurations of network ties, with the presence of linear dominance hierarchies. Event sequences resulting from co-editing of Wikipedia articles are also analyzed in Iba et al. (2010) and in Keegan et al. (2016), but these papers do not link editing events to dominance hierarchies – or the emergence of order in the production of Wikipedia articles. Unlike the majority of existing studies, we do not analyze networks of static or stable dominance relations, but dynamic networks of relational events ordering a sender and a receiver of action. We go beyond counting observed configurations of network ties (or “motifs”), and model the conditional probability of current events on a dyad (*A*,*B*) as a function of its local embeddedness into sequences of past events. We also present new network visualizations that confirm that the empirical estimates produced by the model are consistent with qualitative features of the data. In this paper we present for the first time evidence that linear dominance hierarchies emerge from interaction activities within open social groups observed in a naturalistic (non-experimental) setting over an extended period of time.

### Empirical setting

The opportunity to demonstrate the empirical value of our methodological proposal is presented by data that we have collected on the network of editing events in Wikipedia^1^ – the “free encyclopedia that anyone can edit.” With more than 5 million articles visited each month by more than 500 million unique readers, Wikipedia is perhaps the most comprehensive knowledge repository available and one of the most popular web sites in existence. We focus on a sample of text editing events through which a Wikipedia user deletes parts of the text of Wikipedia articles contributed by another user (Kittur et al. 2007). We interpret these “text undo” events as directed and observable “acts of aggression” – the raw material for the construction of dominance hierarchies (Chase and Seitz 2011).

While apparently distant, concepts of animal ethology that we transpose into the context of open productions are surprisingly relevant for our understanding of the internal organizational dynamics of Wikipedia where conflict among users and contention or disagreement over context are known recurrently to unleash “edit wars” (Yasseri et al. 2012) and to give rise to temporal patterns in the revert network (Tsvetkova et al. 2016).

Status and reputation, rather than overt conflict, are the typical social mechanisms invoked to explain coordination in open productions (Stewart 2005). Reputation ranking in Wikipedia has been studied before by Adler and de Alfaro (2007) and Javanmardi et al. (2010) – among others. These studies have shown that contributions of users with low reputation are more likely to be undone in the future. But we are not aware of studies that have linked individual acts of aggression (as defined above) to the organizational structure of Wikipedia articles. As reported in Sumi et al. (2011) (p. 724 – emphasis added): “[A] way to detect controversy is to view the history of the page, which can show many *war-like acts*, in particular editors reverting the work of other editors.”

In the empirical part of the paper, we concentrate on the network dynamics of these “war-like acts” – and we interpret undo edit events as the “acts of aggression” that behavioral ecologists and animal ethologists have long considered the micro-foundation of dominance hierarchies (Chase and Seitz 2011). The complexity of human societies, in which conflict and collaboration coexist as relational social processes (Simmel 1950), forces us to enrich our analytical framework with the inclusion of “acts of support” through which a user expresses his or her agreement toward another user. This “third party intervention” which we allude to in the title of our paper, takes the form of “redo events” through which a third party re-instates the text that a user had contributed and another user had deleted. Following behavioral ecology, we will call third parties “bystanders” (Chase 1982; Chase et al. 1994; Dugatkin 2001). In network terms, the coexistence of “acts of aggression” and “acts of support” requires the development of models for signed networks – multiplex networks defined in the same set of nodes and that contain both positive as well as negative ties.

Models for signed networks have been adopted to examine co-editing articles (Brandes et al. 2009a), votes for or against requests for adminship (Leskovec et al. 2010), or both (Maniu et al. 2011). The general objective of these works is to analyze patterns of triadic relations in signed networks to confirm or contradict balance theory (Heider 1946; Cartwright and Harary 1956). Adding to this body of work, the model that we introduce in the next section allows us to evaluate more systematically the consistency of dynamic network patterns of negative (undo) and positive (redo) text edits, with linear hierarchy formation at the individual level (degree effects), the dyadic level (reciprocity), and triadic level (closure).

### Hypotheses

**Fig. 1:**
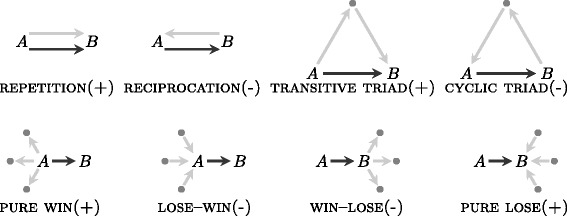
Local configurations of dominance-establishing precursor events (*light gray edges*) predicting future undo edit event from *A* to *B* (*dark gray edges*). *A plus sign* (+) [*minus sign* (-)] indicates the hypothetical increase [decrease] in the probability of observing the next event on (*A*,*B*) given the precursor configuration. These hypotheses are derived from the assumption that undo events or third-party edit events point from higher to lower in the hierarchy (see the text for details). Note that the edges are not binary (present/absent) network ties, but have weights between zero and one

Figure 1 illustrates the different network effects representing the main focus in the analysis that we report in the empirical part of the paper. In Fig. 1, the plus (+) and minus (-) signs indicate how the embedding of the dyad (*A*,*B*) into various configurations of past events (displayed as light-gray edges) is expected to increase (+) or decrease (-) the probability to observe the next relational events on (*A*,*B*). We illustrate the derivation of these hypotheses for dyadic undo events. The derivation for third-party events is very similar. All hypotheses for undo events may be derived from the core hypothesis that an undo event from user *A* directed to user *B* indicates that *A* dominates *B* that is, *A* is likely to be higher in the hierarchy than *B*.

For the core hypothesis of a linear hierarchical order to hold, it has to be the case that a past undo event from *A* to *B*, places *A* above *B* in the hierarchy of text editing relations. In consequence, a future undo event is more likely to be observed again from *A* to *B* than for any random pair of participants (supporting the *inertia*, or dominance REPETITION, hypothesis according to which past undo events within a directed dyad make observing future undo events on the same dyad more likely). Similarly, if a past undo event is observed from *B* to *A*, then *B* is likely to occupy a higher hierarchical position than *A* and, therefore, a future undo event from *A* to *B* is less likely than for a random pair of users (supporting the anti-symmetry hypothesis according to which RECIPROCATION of undo events is unlikely to be observed).

Empirical evidence from studies of animal behavior suggests that individuals losing (winning) one encounter are more likely to lose (win) the next (Chase et al. 1994). Degree-based effects are effects that may be used to characterize pure winners and pure losers (Dugatkin 2001). Pure winners are high outdegree nodes in the text undo network – i. e., participants who delete the text of many others. Pure winners will be high in the dominance hierarchy. A positive and significant PURE WINNER effect would reveal the presence of sequences of dominance events with the same origin node (“source” or “sender”). Pure losers are high indegree nodes in the text undo network – i. e., participants whose text is deleted by many other users. Pure losers will be low in the dominance hierarchy. A positive and significant PURE LOSER effect would reveal the presence of sequences of dominance events with the same destination node (“target” or “receiver”). On the other hand, mixed degree effects in which a previously subordinate participant subsequently dominates others (LOSE–WIN effect), or a previously dominant participant subsequently gets dominated by others (WIN–LOSE effect), are likely to be under-represented in the data (Chase 1982) from which we expect negative effects for these mixed-degree configurations.

Besides dependence on past direct interaction among *A* and *B* and degree effects, a third group of network effects are via indirect interaction, where we hypothesize that dominance establishing events have a tendency towards transitive closure but against cyclic closure. Concretely, if *A* performed many undo events to another user in the past and this same other user enacted many undo events directed at *B*, then *A* is likely to be higher than the other user which, in turn, is higher than *B*; thus, by transitivity, *A* is also higher than *B* and a future undo event on (*A*,*B*) is more likely than on a random pair of users (leading to the hypothetically positive effect of TRANSITIVE TRIAD). On the other hand, if *B* performed many undo events to another user in the past and this same other user enacted many undo events directed at *A*, then *B* is likely to be higher than the other user which, in turn, is higher than *A*; thus, by transitivity, *B* is also higher than *A* and a future undo event on (*A*,*B*) is less likely than on a random pair of users (leading to the hypothetically negative effect of CYCLIC TRIAD).

“Bystanders” are third parties (*C*) observing, but otherwise not directly involved in undo edit events connecting *A* to *B*. As explained by Dugatkin (2001) (p. 348): “When bystander effects are in operation, observers (i. e., bystanders) of aggressive interactions change their assessment of the protagonists’ fighting abilities (depending on who wins and who loses).” In the case of Wikipedia editing network, we use the term “third party edit” to describe a situation whereby user *C* (the bystander) restores text of user *A* that had been undone by user *B*. In this case, the interpretation from the point of view of hierarchy formation is that *A* is higher in the hierarchy than *B* (according to *C*). This hypothetical dominance order induced by third-party edits leads to the same hypotheses for the various configurations from Fig. 1 as dominance induced by undo events (explained above).

## Multiplex networks from text-editing events

### Edit events

We compute relational events sequences encoding dyadic undo, dyadic redo, and third-party editing by successively comparing the text of subsequent revisions of the same Wikipedia article in a similar way as in previous work, e. g., (Adler and de Alfaro 2007; Brandes et al. 2009a; Javanmardi et al. 2010; Maniu et al. 2011; Flöck and Acosta 2014). Building directly on these papers, for each revision we determine which part of the text is newly added, which is deleted, and which previously deleted text is restored by reverting a deletion. We do not treat an edit event as a text modification if large parts of the text (complete sentences in our case) are just moved or duplicated. Large parts of the text are, for instance, moved when a user restructures the page. We do not consider the restructuring user as the author of text that has simply been relocated. We consider a sequence of consecutive revisions by the same user as one revision whose text is that of the last one in the sequence. Such sequences appear when users save their edits several times while editing the page; since we are not interested in self-corrections we can disregard the intermediate steps.

Authorship of text is maintained at the word level. Note that the same word can appear in different places in the text and these different instances can have different authors. Augmenting the computation of edit events proposed in Brandes et al. (2009a), we encode the user interaction resulting from co-editing in a more complete way, as explained in the following.

For each word *w* in the text of each revision we maintain pointers to three potentially different users playing different roles with respect to *w*:

$$[\texttt{author}(w),\texttt{deleter}(w),\texttt{restorer}(w)]\enspace. $$

In this notation author(*w*) is the author who originally added the word *w*. This pointer is set at the revision when the word is added and is never changed afterward. The pointer deleter(*w*) gives the last user who deleted the word. It points to nil when the word is originally added (indicating that no one deleted it so far) and is updated whenever the word is deleted. The pointer restorer(*w*) identifies the last user who added or restored the word. It is set to the author when the word is originally added but, in contrast to author(*w*), the last restorer of a word can change over time when a word is restored after being deleted.

**Fig. 2:**
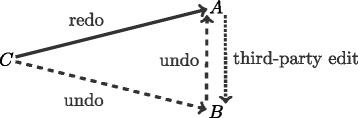
Edit events resulting from a sequence of three revisions where (1) user *A* adds a new word *w*, (2) user *B* deletes *w*, (3) user *C* restores the deleted word *w*. Note that after the third revision, *w* is still authored by *A*, not by the restoring user *C*

To illustrate the definition of the three types of interaction events (dyadic undo, dyadic redo, and third-party edit) we consider the following sequence of three revisions involving three pairwise different users *A*, *B*, and *C* (compare Fig. 2). In Revision 1, user *A* adds a new word *w*. This turns *A* into the author of *w* but it creates no interaction events. In Revision 2, user *B* deletes *w*. This creates an interaction event of type *dyadic undo* from *B* to *A*. In Revision 3, user *C* restores *w*. This creates three interaction events: an event of type *dyadic redo* from *C* to *A*, and event of type *dyadic undo* from *C* to *B*, and an event of type *third-party edit* from *A* to *B*. Note that after the third revision, *w* is still authored by *A*, not by the restoring user *C*.

**Fig. 3:**
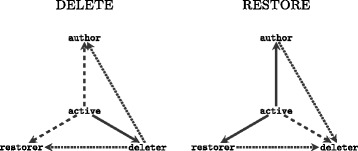
Edit events resulting from the deletion of a word (*left*) and a word being restored (*right*). *Solid lines* encode *dyadic redo* events by which the active user re-does the target user’s edit. *Dashed lines* encode *dyadic undo* events by which the active user makes the target user’s edit undone. *Dotted lines* encode *third-party edit* events by which the active user re-does the source user’s edit that has been made undone by the target user. After deleting a word *w* the user active becomes deleter(*w*) and after restoring *w* user active becomes restorer(*w*). Note that author(*w*) is only set when *w* is originally added and does never change again

In general, interaction events of various types can arise when a word is deleted or restored as illustrated in Fig. 3. Note that we generate a dyadic event only if the active user (i. e., the user who performs the revision) is different from the target of the event and we generate a third-party edit event only if the active user, the source, and the target are three pairwise different users.

Concretely, when the active user *C* deletes a word *w* then this creates a event of type undo on the dyad (*C*,*A*) – where *A* is the author of *w* – and another event of type undo on the dyad (*C*,*R*) – where *R* is the last restorer of *w*. The deletion of *w* further creates an event of type redo on the dyad (*C*,*D*) – where *D* is the last deleter of *w* – *if* the word *w* has ever been deleted before. The deletion of *w* creates an event of type *third-party edit* on the dyad (*D*,*A*), expressing that the active user *C* favors the deleter’s (*D*) edits over those of the original author (*A*). A deletion creates an event of type third-party edit on the dyad (*D*,*R*), expressing that the active user *C* favors the deleter’s (*D*) edits over those of the last restorer (*R*). When the active user *C* restores a (previously deleted) word *w* then this creates a event of type redo on the dyad (*C*,*A*) and another event of type redo on the dyad (*C*,*R*). Restoring *w* further creates an event of type undo on the dyad (*C*,*D*). Moreover, restoring *w* creates an event of type third-party edit on the dyad (*A*,*D*), expressing that the active user *C* favors the author’s (*A*) edits over those of the deleter (*D*) and it creates an event of type third-party edit on the dyad (*R*,*D*), expressing that the active user *C* favors the last restorer’s (*R*) edits over those of the last deleter (*D*).

When several words are deleted or restored in one revision, we separately aggregate events of the same type that have the same source and target; these events have weight equal to the number of words modified in that manner. Thus, the revision *r*_*i*_ uploaded by the active user *C* gives rise to the following (integer) values.

$$\begin{array}{@{}rcl@{}} \forall_{A\not=C}\colon\;undo_{i}(C,A)&=&\text{number of undo events in \(r_{i}\) on \((C,A)\)}\\ \forall_{A\not=C}\colon\;redo_{i}(C,A)&=&\text{number of redo events in \(r_{i}\) on \((C,A)\)}\\ \forall_{A\not=C\not=B\not=A}\colon\;tpe_{i}(A,B)&=&\text{num.\ of third-party edit events in \(r_{i}\) on \((A,B)\)} \end{array} $$

Note that many of these values might be zero for any given revision *r*_*i*_.

### The event potential

While iterating over the revisions of an article we do not only consider events that happen, but also the set of *potential* events that *could have*, but *did not necessarily* happen in the respective revision. More precisely, we keep track for each user *A* and for each of the dyadic event types *x* (that is, dyadic undo and dyadic redo) how many events of type *x* can have target *A* in revision *r*_*i*_. If *C* is the active user of revision *r*_*i*_, then the event potential for dyadic undo with target *A*, denoted by *p* *o* *t*.*u* *n* *d* *o*_*i*_(*C*,*A*), is the number of words that are currently (that is, just before revision *r*_*i*_) in the article of which *A* is the author plus the number of words that are currently in the article of which *A* is the last restorer plus the number of words that are currently not in the article of which *A* is the last deleter. The event potential for dyadic redo with target *A*, denoted by *p* *o* *t*.*r* *e* *d* *o*_*i*_(*C*,*A*), is similarly obtained by substituting the words “in the article” with “not in the article”. For convenience we define *p* *o* *t*.*u* *n* *d* *o*_*i*_(*C* ^′^,*A*)=0 and *p* *o* *t*.*r* *e* *d* *o*_*i*_(*C* ^′^,*A*)=0 for every *C* ^′^ that is not the active user of revision *r*_*i*_ and we define *p* *o* *t*.*u* *n* *d* *o*_*i*_(*C*,*C*)=0 and *p* *o* *t*.*r* *e* *d* *o*_*i*_(*C*,*C*)=0, since we do not consider self-correction.

Likewise, for each ordered pair of different users (*A*,*B*) we keep track of the potential for third-party edit events which a user *C* (different from *A* and from *B*) can assign to the dyad (*A*,*B*) in revision *r*_*i*_. This value, denoted by *p* *o* *t*.*t* *p* *e*_*i*_(*A*,*B*), is the number of words that are currently (that is, just before revision *r*_*i*_) in the article of which *A* is the last deleter and *B* is the author, plus the number of words that are currently in the article of which *A* is the last deleter and *B* is the last restorer, plus the number of words that are currently not in the article of which *A* is the author and *B* is the last deleter, plus the number of words that are currently not in the article of which *A* is the last restorer and *B* is the last deleter. Note that *p* *o* *t*.*t* *p* *e*_*i*_(*A*,*B*) is zero if the revision *r*_*i*_ is performed by user *A* or user *B* and it is zero if *A*=*B*.

### The network of past events

While iterating over the sequence of revisions of a article, we successively update six functions (called *dyad-level attributes*) defined on ordered pairs (*A*,*B*) of different users and taking integer values. Three of these attributes (the *cumulative events*) count events of the three types that actually happened on (*A*,*B*) in any of the revisions *r*_1_,…,*r*_*i*_ and three of them (the *cumulative event potentials*) add up the number of events (of the three types) that could have happened on (*A*,*B*) in any of the revisions *r*_1_,…,*r*_*i*_.

Thus, for each different pair of users (dyads) *A* and *B*, the attribute *cumulative dyadic undo* on (*A*,*B*) after revision *r*_*i*_, denoted by *c* *u* *m*.*u* *n* *d* *o*_*i*_(*A*,*B*), is the sum of the values of the actually observed past events *u* *n* *d* *o*_*j*_(*A*,*B*), *j*=1,…,*i*, that is

$$cum.undo_{i}(A,B)=\sum\limits_{j=1}^{i}undo_{j}(A,B)\enspace. $$

Likewise, the attribute *cumulative dyadic undo potential* on (*A*,*B*) after revision *r*_*i*_, denoted by *c* *u* *m*.*p* *o* *t*.*u* *n* *d* *o*_*i*_(*A*,*B*), is the sum of the potentials by which *B* could be the target of dyadic undo events at revisions *r*_*j*_,*j*=1,…,*i*, that is

$$cum.pot.undo_{i}(A,B)=\sum\limits_{j=1}^{i}pot.undo_{j}(A,B)\enspace. $$

The definition of the two attributes *cumulative dyadic redo* and *cumulative dyadic redo potential* is done accordingly, that is

$$\begin{array}{@{}rcl@{}} cum.redo_{i}(A,B)&=&\sum\limits_{j=1}^{i}redo_{j}(A,B)\\ cum.pot.redo_{i}(A,B)&=&\sum\limits_{j=1}^{i}pot.redo_{j}(A,B)\enspace. \end{array} $$

The attributes *cumulative third-party edit* and *cumulative third-party edit potential* on a pair of different users (*A*,*B*) are defined accordingly:

$$\begin{array}{@{}rcl@{}} cum.tpe_{i}(A,B)&=&\sum\limits_{j=1}^{i}tpe_{j}(A,B)\\ cum.pot.tpe_{i}(A,B)&=&\sum\limits_{j=1}^{i}pot.tpe_{j}(A,B)\enspace. \end{array} $$

Finally, to describe the past interaction on dyads (*A*,*B*) we consider, separately for the three event types, the ratio of actually observed events divided by the cumulative potential for such events.^2^ Thus it is

$$\begin{array}{@{}rcl@{}} ratio.undo_{i}(A,B)&=&cum.undo_{i}(A,B)/cum.pot.undo_{i}(A,B)\\ ratio.redo_{i}(A,B)&=&cum.redo_{i}(A,B)/cum.pot.redo_{i}(A,B)\\ ratio.tpe_{i}(A,B)&=&cum.tpe_{i}(A,B)/cum.pot.tpe_{i}(A,B) \end{array} $$

These rations are defined on the closed interval [0,1] (including the boundaries of the set). For instance, the past dyadic undo ratio, *r* *a* *t* *i* *o*.*u* *n* *d* *o*_*i*_(*A*,*B*), is the number of words of *B* that are undone by *A* in one of the revisions *r*_1_,…,*r*_*i*_, divided by the number of words of *B* that could have been made undone by *A* in one of these revisions. Similar interpretations apply to past dyadic redo ratio and past third-party edit ratio. For many applications, these ratios are more informative than the unnormalized counts *c* *u* *m*.*u* *n* *d* *o*_*i*_(*A*,*B*) etc. Indeed, the information that *A* (say) deleted 10 of *B*’s words is hard to interpret if we ignore how much text of *B* could have been deleted by *A*. If *B* authored (say) 1,000 words in the article’s text and *A* deleted only 10 words of it, then this indicates a minor rewriting but not a major disagreement. If on the other hand, the 10 deleted words constituted all of *B*’s text, then it reveals that *A* erased the entire contribution of *B*. In the statistical models introduced in the next section we construct explanatory variables based on these ratios.

## Statistical models for relational events

### Outcome variables.

Whenever a revision *r*_*i*_ is performed by a user *A*, then *A* can potentially initiate *p* *o* *t*.*u* *n* *d* *o*_*i*_(*A*,*B*) undo events towards any target user *B*≠*A* and *A* can potentially initiate *p* *o* *t*.*r* *e* *d* *o*_*i*_(*A*,*B*) redo events towards any target user *B*≠*A*. Likewise, *A* has the potential to initiate *p* *o* *t*.*t* *p* *e*_*i*_(*B*,*C*) third-party edit events on any dyad (*B*,*C*), where *B*≠*A*≠*C*≠*B*. We want to model the probabilities that *A* indeed initiates these potential events. Thus, the three outcome variables that we consider are the numbers of events of a given type and a given target user actually performed in revision *r*_*i*_ divided by the respective potential for such events, that is, our model explains the observed probabilities

$$\begin{array}{@{}rcl@{}} prob.undo_{i}(A,B)&=&undo_{i}(A,B)/pot.undo_{i}(A,B)\\ prob.redo_{i}(A,B)&=&redo_{i}(A,B)/pot.redo_{i}(A,B)\\ prob.tpe_{i}(A,B)&=&tpe_{i}(A,B)/pot.tpe_{i}(A,B) \end{array} $$

which are defined for any user *B* for which the respective denominator is not zero (note that if the denominator is zero, then we will not observe such an event on the given dyad).

For each event type we use a binomial model where instances are words that may be potentially changed, a “success” instance is such a word that is actually changed in the revision, and a “failure” instance is such a word that is left unchanged. The probability that a potential change occurs is specified in logistic regression models

$$\log\left(\frac{p}{1-p}\right)=\theta\cdot s(i;A,B)\enspace, $$

where *p* is one of *p* *r* *o* *b*.*u* *n* *d* *o*_*i*_(*A*,*B*), *p* *r* *o* *b*.*r* *e* *d* *o*_*i*_(*A*,*B*), or *p* *r* *o* *b*.*t* *p* *e*_*i*_(*A*,*B*); $\theta \in \mathbb {R}^{k}$ is a vector of parameters to be estimated; and $s(i;A,B)\in \mathbb {R}^{k}$ is a vector of explanatory variables (*statistics*) characterizing the embedding of the dyad (*A*,*B*) into the network of past events at revision *r*_*i*_, introduced below.

### Explanatory variables

When modeling the probability of change events that could happen in revision *r*_*i*_, we use only information about past interaction resulting from revisions *r*_1_,…,*r*_*i*−1_, that is, revisions that happened strictly before *r*_*i*_. These explanatory variables are defined by combinations of three dyadic attributes (past dyadic undo ratio, past dyadic redo ratio, and past third-party edit ratio) on the configurations shown in Fig. 1. More precisely, we use the following explanatory variables to explain events in revision *r*_*i*_ (where for readability we write *x* instead of *r* *a* *t* *i* *o*.*u* *n* *d* *o*):

$$\begin{array}{@{}rcl@{}} undo.repetition_{i}(A,B)&=&x_{i-1}(A,B)\\ undo.reciprocation_{i}(A,B)&=&x_{i-1}(B,A)\\ undo.outdeg.src_{i}(A,B)&=&\sum\limits_{B'\not=A}x_{i-1}(A,B')\\ undo.indeg.src_{i}(A,B)&=&\sum\limits_{B'\not=A}x_{i-1}(B',A)\\ undo.outdeg.trg_{i}(A,B)&=&\sum\limits_{A'\not=B}x_{i-1}(B,A')\\ undo.indeg.trg_{i}(A,B)&=&\sum\limits_{A'\not=B}x_{i-1}(A',B)\\ undo.transitive_{i}(A,B)&=&\sqrt{\sum\limits_{C\not=A,B}x_{i-1}(A,C)\cdot x_{i-1}(C,B)}\\ undo.cyclic_{i}(A,B)&=&\sqrt{\sum\limits_{C\not=A,B}x_{i-1}(B,C)\cdot x_{i-1}(C,A)} \end{array} $$

In the notation above *deg* stands for “degree,” *src* for “source,” and *trg* for “target.”

The explanatory variables based on past redo events or past third-party edit events are defined by the same formulas by substituting *x* for *r* *a* *t* *i* *o*.*r* *e* *d* *o* or for *r* *a* *t* *i* *o*.*t* *p* *e*, respectively.

For undo events and for third-party edit events Table 1 lists the correspondence between the names of explanatory variables based on degrees and the network effects mentioned in Fig. 1 (the names of the other variables match the names of the corresponding effects rather directly).

**Table 1:** Correspondence between variable names and names of degree effects (compare Fig. 1) for undo events and third-party edit events

Variable name	Effect name
*outdeg.src*	pure win
*indeg.src*	lose–win
*outdeg.trg*	win–lose
*indeg.trg*	pure lose

To simplify comparison of different effects we normalize the explanatory variables by their standard deviation. This operation makes it easier to compare effects that may otherwise be of very different magnitude. Since average probabilities are very close to zero (cf. Table 2), we can interpret the estimated parameters in the following intuitive (if not formally correct) way: if we estimated a parameter *θ* for the variable *x* when modeling the (say) dyadic undo probability *p*, then (hypothetically) increasing *x* by one standard deviation (that is by 1) multiplies the probability *p* by exp(*θ*). For instance, a parameter *θ*=0.1 implies a probability-increase by about 10.5%, a parameter *θ*=0.5 implies a probability-increase by about 65%, when *x* is increased by one.

**Table 2:** Number of instances and non-null instances in the analyzed data

	Dyadic undo	Dyadic redo	Third-party edit
No. potential dyads	3,126,047	1,753,160	4,852,052
No. non-null dyads	37,823	21,411	21,335
Dyad-density	1.21%	1.22%	0.44%
No. potential words	361,673,769	359,365,077	348,420,292
No. changed words	1,738,728	785,233	783,190
Word-change density	0.48%	0.22%	0.23%

### Research design and data

We analyzed the histories of a sample of ten articles from the English-language Wikipedia, chosen uniformly at random from the set of articles that have at least 1,000 revisions.^3^ In March 2016 there were 56,042 articles (pages in the main namespace that are not redirects) with at least a thousand revisions. (Altogether there are about 5 million articles; the mean number of revisions per article is just 86.) The ten sampled articles have together 12,719 revisions (disregarding successive revisions by the same user) performed by 7,657 different users.

We note that our number of observations is not just ten since the unit of analysis is not the article but the dyadic event. Table 2 reports the number of dyad-timepoints on which there could have happened an event of the various types, the number of actual dyadic events, the number of words that could have been modified, and the number of actual word modifications. The approach to analyze 10 random articles (rather than just one) has been chosen since it reduces the likelihood of accidentally analyzing an article with an unusual structure. The restriction to articles with at least a thousand revisions is motivated by the consideration that hierarchy formation takes some time, and is more meaningful when the number of users is not too small. What increases the runtime of our analysis is that we consider not only the actually occurring events but also those that could have happened. However, we strongly believe that this is necessary since an observation such as “user *A* deleted 10 of user *B*’s words” is meaningless if we disregard how many of *B*’s words user *A* did not touch and/or if we disregard all the other users with which *A* potentially could have interacted but did not. The results reported in the next section have been estimated to maximize the joint likelihood of all events from all sampled articles.

## Results

### Dyad-level effects

Table 3 reports logistic regression parameters explaining the probability of dyadic undo events by past interaction on the same dyad and the reverse dyad.

**Table 3:** Explaining dyadic undo by past dyadic undo and past dyadic redo on the same dyad

	Dyad model	Dyadic repetition	Dyadic reciprocation
(Intercept)	−5.427 (0.001)^∗∗∗^	−5.427 (0.001)^∗∗∗^	−5.427 (0.001)^∗∗∗^
undo.repetition	0.222 (0.000)^∗∗∗^	0.115 (0.000)^∗∗∗^	
redo.repetition	−0.288 (0.002)^∗∗∗^	−0.071 (0.003)^∗∗∗^	
undo.reciprocation	0.060 (0.000)^∗∗∗^		−0.064 (0.000)^∗∗∗^
redo.reciprocation	−0.093 (0.000)^∗∗∗^		0.030 (0.001)^∗∗∗^
undirected.undo		0.127 (0.000)^∗∗∗^	0.262 (0.000)^∗∗∗^
undirected.redo		−0.243 (0.001)^∗∗∗^	−0.321 (0.003)^∗∗∗^
AIC	17,531,308.231	17,531,308.231	17,531,308.231
Num. obs.	3,126,047	3,126,047	3,126,047

^∗∗∗^ *p*<0.001

In the first model, we observe that past dyadic undo on (*A*,*B*) increases (positive parameter for undo.repetition) the probability of future dyadic undo on (*A*,*B*) – providing evidence of repeated winning over the same target (as predicted in our hypotheses). However, we see that past dyadic undo on the reverse dyad (*B*,*A*) also increases the probability of dyadic undo on (*A*,*B*). Thus, users that receive an undo event have a tendency to fight back hindering hierarchy formation and running counter to our anti-reciprocation hypothesis. Likewise, we see that past redo on (*A*,*B*) reduces the probability of dyadic undo on (*A*,*B*) (as expected). However, past redo on (*B*,*A*) also reduces the probability of dyadic undo on (*A*,*B*). This makes the (hypothetical) interpretation that redo events tend to go from lower to higher in the hierarchy questionable.

Looking more closely at parameter size, we see that a dyadic undo event on (*A*,*B*) has two effects. First it increases the future hostility (likelihood of undo events) on (*A*,*B*) and on (*B*,*A*). This is inconsistent with a hierarchical interpretation of undo events going from higher to lower, but is consistent with a retaliation or polarization interpretation to be discussed in the conclusion. A similar finding has been made by (Leskovec et al. 2010) who analyzed voting behavior of Wikipedians. A second effect, however, is that a dyadic undo event on (*A*,*B*) increases the future undo probability on (*A*,*B*) more than on (*B*,*A*), thereby increasing the relative dominance of (*A*,*B*) over (*B*,*A*), if undo events establish a dominance order. This second effect becomes more apparent if we control for the increase in undo activity on both dyads (*A*,*B*) and (*B*,*A*) by defining a variable

$$undirected.undo_{i}(A,B)=undo.repetition_{i}(A,B)+undo.recipr_{i}(A,B) $$

which is the past dyadic undo ratio on (*A*,*B*) plus the past dyadic undo ratio on (*B*,*A*) (after taking the sum we normalize this variable to standard deviation one). We define a variable *u* *n* *d* *i* *r* *e* *c* *t* *e* *d*.*r* *e* *d* *o*_*i*_(*A*,*B*) accordingly. In the second and third model in Table 3 we see that, controlling for the undirected increase in undo activity, an undo event on (*A*,*B*) increases the future undo probability on (*A*,*B*) more than expected and that it increases the future undo probability on (*B*,*A*) less than expected. A similar result is obtained for dyadic redo, where a redo event on (*A*,*B*)*decreases* the future undo probability on (*A*,*B*) more than expected and that on (*B*,*A*) less than expected. We note that the three models in Table 3 are equivalent since their variables are linear transformations of each other.

In summary, a dyadic undo event on (*A*,*B*) has two effects: a polarization or retaliation effect (also consistent with structural balance theory (Heider 1946)) increasing the hostility level on the undirected dyad {*A*,*B*} and a hierarchical effect that shifts the relative dominance towards the direction (*A*,*B*). It is likely that the experimentally found anti-symmetry of dominance events among chicken (e. g., (Chase 1982)) is due to the small network size. In larger and therefore sparser networks it is likely that reciprocation of acts of dominance, albeit rare, might occur with a higher probability than the low baseline probability of interacting at all.

We obtain similar findings when estimating the probability of dyadic redo by dyadic effects (with the understanding that redo events hypothetically point from lower to higher in the hierarchy, similar to the argument from (Leskovec et al. 2010)); see Table 4.

**Table 4:** Explaining dyadic redo by past dyadic undo and past dyadic redo on the same dyad

	Dyad model	Dyadic repetition	Dyadic reciprocation
(Intercept)	−6.206 (0.001)^∗∗∗^	−6.206 (0.001)^∗∗∗^	−6.206 (0.001)^∗∗∗^
undo.repetition	−0.114 (0.001)^∗∗∗^	−0.091 (0.002)^∗∗∗^	
redo.repetition	0.332 (0.000)^∗∗∗^	0.180 (0.001)^∗∗∗^	
undo.reciprocation	−0.008 (0.001)^∗∗∗^		0.031 (0.001)^∗∗∗^
redo.reciprocation	0.085 (0.000)^∗∗∗^		−0.100 (0.000)^∗∗∗^
undirected.undo		−0.024 (0.002)^∗∗∗^	−0.123 (0.002)^∗∗∗^
undirected.redo		0.180 (0.000)^∗∗∗^	0.390 (0.001)^∗∗∗^
AIC	8,923,544.712	8,923,544.712	8,923,544.712
Num. obs.	1,753,160	1,753,160	1,753,160

^∗∗∗^ *p*<0.001

Table 5 reports logistic regression parameters for the probability of third-party edit events by past third-party edits on the same and the reverse dyad. In contrast to dyadic interaction, we see that third-party edits are clearly anti-reciprocal: controlling for the undirected increase in the event probability is here not necessary although it strengthens the anti-symmetry. This means that if a different user *C* restores *A*’s edits that had been undone by *B*, then the probability that *C* (or any other user different from *A* and *B*) later reverses this order decreases (negative parameter for *tpe.reciprocation*) and the probability that *C* (or any other user different from *A* to *B*) initiates a future third-party edit event in the same direction increases (positive parameter for *tpe.repetition*). Thus, third-party edit events are more consistent with the hierarchical interpretation than dyadic interaction. Apparently bystanders can adjudicate the dominance order among *A* and *B* more reliably than *A* or *B* themselves. This finding could also be explained by the reasoning that third users are more likely to make edits based on relative quality evaluations (compare our discussion in the conclusion) and/or less likely to retaliate since it is not “their” text that has been deleted previously.

**Table 5:** Explaining third-party edit events by past third-party edits on the same dyad

	Dyad model	Dyadic repetition	Dyadic reciprocation
(Intercept)	−6.196 (0.001)^∗∗∗^	−6.196 (0.001)^∗∗∗^	−6.196 (0.001)^∗∗∗^
tpe.repetition	0.379 (0.000)^∗∗∗^	0.391 (0.001)^∗∗∗^	
tpe.reciprocation	−0.022 (0.001)^∗∗∗^		−0.740 (0.001)^∗∗∗^
undirected.tpe		−0.025 (0.001)^∗∗∗^	0.814 (0.001)^∗∗∗^
AIC	9,721,545.325	9,721,545.325	9,721,545.325
Num. obs.	4,852,052	4,852,052	4,852,052

^∗∗∗^ *p*<0.001

### Degree effects

Table 6 reports estimated parameters of models explaining the probability of dyadic undo on (*A*,*B*) by past interaction on edges incident to *A* (source) and *B* (target). In the first model (“degree model”) we find some effects consistent with the hierarchical interpretation. The positive parameter of *undo.oudeg.src* implies that users who initiated many undo events directed to any user are more likely to initiate future undo events (what we called PURE WINNER effect). The positive parameter of *undo.indeg.trg* implies that users who received many undo events initiated by any user are more likely to have their edits undone in the future (PURE LOSER effect). The negative parameter of *undo.indeg.src* implies that users who received many undo events in the past are less likely to initiate undo events in the future (MIXED LOSE-WIN effect). While these three findings support our hypotheses about degree effects, we can also find effects inconsistent with the hierarchical interpretation of undo events: the positive parameter of *undo.outdeg.trg* implies that users who initiated many undo events in the past are more likely to receive undo events in the future (MIXED WIN-LOSE effect). This is against the hierarchical interpretation and rather points to generalized reciprocation for undo events: users who initiate a lot of undo events are likely to have their edits undone by (potentially) other users.

**Table 6:** Explaining dyadic undo by past undo and redo on dyads incident to source and target (degree effects)

	Degree model	Generalized inertia	Generalized reciprocity
(Intercept)	−5.608 (0.001)^∗∗∗^	−5.608 (0.001)^∗∗∗^	−5.608 (0.001)^∗∗∗^
undo.outdeg.src	0.152 (0.001)^∗∗∗^	0.178 (0.004)^∗∗∗^	
redo.outdeg.src	0.125 (0.001)^∗∗∗^	−1.933 (0.006)^∗∗∗^	
undo.indeg.src	−0.004 (0.001)^∗∗∗^		−0.028 (0.001)^∗∗∗^
redo.indeg.src	0.263 (0.001)^∗∗∗^		0.247 (0.001)^∗∗∗^
undo.outdeg.trg	0.205 (0.000)^∗∗∗^		−0.317 (0.001)^∗∗∗^
redo.outdeg.trg	−0.054 (0.000)^∗∗∗^		2.088 (0.005)^∗∗∗^
undo.indeg.trg	0.495 (0.001)^∗∗∗^	0.301 (0.001)^∗∗∗^	
redo.indeg.trg	−0.920 (0.002)^∗∗∗^	−0.897 (0.002)^∗∗∗^	
undo.degree.src		−0.027 (0.004)^∗∗∗^	0.161 (0.001)^∗∗∗^
redo.degree.src		2.203 (0.006)^∗∗∗^	0.134 (0.001)^∗∗∗^
undo.degree.trg		0.280 (0.000)^∗∗∗^	0.712 (0.002)^∗∗∗^
redo.degree.trg		−0.061 (0.001)^∗∗∗^	−2.402 (0.005)^∗∗∗^
AIC	15,338,717.559	15,338,717.559	15,338,717.559
Num. obs.	3,126,047	3,126,047	3,126,047

^∗∗∗^ *p*<0.001

Findings on the degree effects of redo events on future undo events are mixed: the parameters of *redo.indeg.src* (positive) and *redo.outdeg.trg* (negative) have the expected signs but the parameters of *redo.outdeg.src* (postive) and *redo.indeg.trg* (negative) point in the unexpected direction.

As in the case of dyad effects, the effects of the directed degree variables (for undo events and for redo events) become consistent with the hierarchical interpretation once we control for the *undirected* degrees (see the models “generalized inertia” and “generalized reciprocity” in Table 6). The variable *undo.degree.src* is defined to be the sum of *undo.outdeg.src* with *undo.indeg.src* and measures how much the source actor is involved in undo events – outgoing or incoming. The variables *redo.degree.src*, *undo.degree.trg*, and *redo.degree.trg* are obtained accordingly by summing out-degrees and in-degrees.

We also controlled for the dyadic effects from Table 3 in the degree models (results are not reported in this paper) which did not change the findings qualitatively.

Table 7 reports estimated parameters for models explaining the probability of dyadic redo events by degree effects. In the first model (“degree model”) the parameters of *undo.outdeg.src* (negative), *redo.outdeg.src* (positive), *undo.indeg.trg* (negative), and *redo.indeg.trg* (positive) have the predicted sign. However, the parameters of *undo.indeg.src* (negative), *redo.indeg.src* (positive), *undo.outdeg.trg* (negative), and *redo.outdeg.trg* (positive) have the opposite sign as predicted by out hypotheses. In contrast to the model explaining dyadic undo events, controlling for the undirected degrees (models “generalized inertia” and “generalized reciprocity” in Table 7) does not turn the parameters of all variables in the predicted direction (rather it creates new inconsistencies). Together, the findings reported in Table 7 make the hierarchical interpretation of dyadic redo pointing from lower to higher in the hierarchy more questionable.

**Table 7:** Explaining dyadic redo by past undo and redo on dyads incident to source and target (degree effects)

	Degree model	Generalized inertia	Generalized reciprocity
(Intercept)	−5.624 (0.002)^∗∗∗^	−5.624 (0.002)^∗∗∗^	−5.624 (0.002)^∗∗∗^
undo.outdeg.src	−0.267 (0.002)^∗∗∗^	0.340 (0.011)^∗∗∗^	
redo.outdeg.src	0.273 (0.001)^∗∗∗^	−0.828 (0.011)^∗∗∗^	
undo.indeg.src	−0.101 (0.002)^∗∗∗^		−0.056 (0.002)^∗∗∗^
redo.indeg.src	0.139 (0.001)^∗∗∗^		0.104 (0.001)^∗∗∗^
undo.outdeg.trg	−0.253 (0.000)^∗∗∗^		−0.106 (0.002)^∗∗∗^
redo.outdeg.trg	0.137 (0.000)^∗∗∗^		−1.099 (0.003)^∗∗∗^
undo.indeg.trg	−0.077 (0.001)^∗∗∗^	0.055 (0.001)^∗∗∗^	
redo.indeg.trg	0.506 (0.001)^∗∗∗^	0.450 (0.001)^∗∗∗^	
undo.degree.src		−0.646 (0.011)^∗∗∗^	−0.284 (0.002)^∗∗∗^
redo.degree.src		1.181 (0.012)^∗∗∗^	0.293 (0.001)^∗∗∗^
undo.degree.trg		−0.282 (0.000)^∗∗∗^	−0.164 (0.003)^∗∗∗^
redo.degree.trg		0.153 (0.000)^∗∗∗^	1.378 (0.003)^∗∗∗^
AIC	8,438,636.039	8,438,636.039	8,438,636.039
Num. obs.	1,753,160	1,753,160	1,753,160

^∗∗∗^ *p*<0.001

Table 8 reports estimated parameters of models explaining third-party edit events by degree effects. Three of the effects in the first model (“degree model”) are consistent with the hierarchical interpretation of third-party edit events pointing from higher to lower hierarchical positions: the positive parameter of *tpe.outdeg.src* implies that dominant actors continue to dominate (PURE WINNER effect), the negative parameter of *tpe.outdeg.trg* implies that dominant actors tend *not* to get dominated, and the positive parameter of *tpe.indeg.trg* implies that dominated actors tend to get dominated (PURE LOSER effect). The exception is a positive effect of *tpe.indeg.src* which suggests that subordinates are more likely to dominate in the future (MIXED LOSE-WIN effect). Controlling for the undirected degrees (models “generalized inertia” and “generalized reciprocity” in Table 8) brings all degree effects in accordance with the hierarchical interpretation. Controlling for the dyadic effects from Table 5 in the degree model (not reported in this paper) yields qualitatively the same findings.

**Table 8:** Explaining third-party edit events by past third-party edits incident to source and target (degree effects)

	Degree model	Generalized inertia	Generalized reciprocity
(Intercept)	−4.994 (0.002)^∗∗∗^	−4.994 (0.002)^∗∗∗^	−4.994 (0.002)^∗∗∗^
tpe.outdeg.src	0.293 (0.001)^∗∗∗^	0.064 (0.001)^∗∗∗^	
tpe.indeg.src	0.166 (0.000)^∗∗∗^		−0.046 (0.001)^∗∗∗^
tpe.outdeg.trg	−2.967 (0.004)^∗∗∗^		−3.656 (0.004)^∗∗∗^
tpe.indeg.trg	0.407 (0.001)^∗∗∗^	2.158 (0.002)^∗∗∗^	
tpe.degree.src		0.275 (0.000)^∗∗∗^	0.352 (0.001)^∗∗∗^
tpe.degree.trg		−3.396 (0.005)^∗∗∗^	0.789 (0.001)^∗∗∗^
AIC	8,233,430.699	8,233,430.699	8,233,430.699
Num. obs.	4,852,052	4,852,052	4,852,052

^∗∗∗^ *p*<0.001

### Triad-level effects

Table 9 reports logistic regression parameters explaining the probability of undo events on a dyad (*A*,*B*) by past interaction on two-paths of the form (*A*,*C*),(*C*,*B*), forming a transitive triad, and on two-paths of the form (*B*,*C*),(*C*,*A*), forming a cyclic triad. The first model (“triad model”) reveals that the embedding of (*A*,*B*) in an undo two-path increases the probability of an undo event on (*A*,*B*), irrespective of whether the resulting triad is transitive (consistent with the hierarchical interpretation) or cyclic (inconsistent with this interpretation). Likewise, the embedding of (*A*,*B*) in a redo two-path decreases the probability of an undo event on (*A*,*B*), irrespective of whether the resulting triad is transitive (consistent with our hypothesis) or cyclic (inconsistent with our hypothesis).

**Table 9:** Explaining dyadic undo by past undo and redo on transitive and cyclic two-paths

	Triad model	Transitive triad	Cyclic triad
(Intercept)	−5.264 (0.001)^∗∗∗^	−5.264 (0.001)^∗∗∗^	−5.264 (0.001)^∗∗∗^
undo.transitive	0.149 (0.001)^∗∗∗^	0.049 (0.001)^∗∗∗^	
redo.transitive	−0.362 (0.002)^∗∗∗^	−0.045 (0.003)^∗∗∗^	
undo.cyclic	0.027 (0.000)^∗∗∗^		−0.013 (0.000)^∗∗∗^
redo.cyclic	−0.135 (0.001)^∗∗∗^		0.019 (0.001)^∗∗∗^
undo.twopath		0.105 (0.001)^∗∗∗^	0.156 (0.001)^∗∗∗^
redo.twopath		−0.355 (0.002)^∗∗∗^	−0.405 (0.003)^∗∗∗^
AIC	18,958,234.850	18,958,234.850	18,958,234.850
Num. obs.	3,126,047	3,126,047	3,126,047

^∗∗∗^ *p*<0.001

Controlling for undo two-paths in any direction (variable *undo.twopath* defined as the sum of *undo.transitive* and *undo.cyclic*) and for redo two-paths in any direction (variable *redo.twopath* defined as the sum of *redo.transitive* and *redo.cyclic*) reveals a preference for transitive over cyclic closure of undo events—consistent with the hierarchical interpretation. Controlling for dyad effects and degree effects (not reported in this paper), however, does *not* keep the triadic effects for undo events stable.

Table 10 reports logistic regression parameters explaining the probability of redo events on a dyad (*A*,*B*) by past interaction on two-paths of the form (*A*,*C*),(*C*,*B*), forming a transitive triad, and on two-paths of the form (*B*,*C*),(*C*,*A*), forming a cyclic triad. The first model (“triad model”) reveals that the embedding of (*A*,*B*) in an undo two-path or in a redo two-path increases the probability of an redo event on (*A*,*B*), irrespective of whether the resulting triad is transitive (for transitive redo two-paths this is consistent with our hypothesis but for transitive undo two-paths it is inconsistent with our hypothesis) or cyclic (for cyclic redo two-paths this is inconsistent with our hypothesis but for cyclic undo two-paths it is consistent with our hypothesis).

**Table 10:** Explaining dyadic redo by past undo and redo on transitive and cyclic two-paths

	Triad model	Transitive triad	Cyclic triad
(Intercept)	−6.247 (0.001)^∗∗∗^	−6.247 (0.001)^∗∗∗^	−6.247 (0.001)^∗∗∗^
undo.transitive	0.062 (0.001)^∗∗∗^	−0.094 (0.001)^∗∗∗^	
redo.transitive	0.333 (0.001)^∗∗∗^	0.201 (0.001)^∗∗∗^	
undo.cyclic	0.039 (0.000)^∗∗∗^		0.023 (0.000)^∗∗∗^
redo.cyclic	0.054 (0.000)^∗∗∗^		−0.082 (0.001)^∗∗∗^
undo.twopath		0.161 (0.001)^∗∗∗^	0.064 (0.001)^∗∗∗^
redo.twopath		0.146 (0.001)^∗∗∗^	0.369 (0.001)^∗∗∗^
AIC	8,936,083.764	8,936,083.764	8,936,083.764
Num. obs.	1,753,160	1,753,160	1,753,160

^∗∗∗^ *p*<0.001

Controlling for undo two-paths in any direction and for redo two-paths in any direction reveals a preference for transitive over cyclic closure of redo events—consistent with the hierarchical interpretation.

Table 11 reports logistic regression parameters explaining the probability of third-party edit events on (*A*,*B*) by past interaction on two-paths of the form (*A*,*C*),(*C*,*B*), forming a transitive triad, and on two-paths of the form (*B*,*C*),(*C*,*A*), forming a cyclic triad. The first model (“triad model”) reveals that past third-party edits on two paths connecting *A* and *B* decrease the probability of future third-party edits on the dyad (*A*,*B*) irrespective of the direction of these two-paths. For transitive two-paths, this contradicts the hierarchical interpretation and for cyclic it is consistent with our hypothesis. Note that both findings are consistent with a polarization interpretation of dominance ties, discussed later in the conclusion (an enemy of an enemy is not an enemy). When we control for the dominance-reducing effect of undirected two-paths (models “transitive triad” and “cyclic triad” in Table 11), we find a prevalence of transitive over cyclic closure (consistent with the hierarchical interpretation. As for dyadic undo events, controlling for dyad and degree effects (not reported in this paper) does *not* keep these triadic effects stable.

**Table 11:** Explaining third-party edit events by past third-party edits on transitive and cyclic two-paths

	Triad model	Transitive triad	Cyclic triad
(Intercept)	−5.403 (0.001)^∗∗∗^	−5.403 (0.001)^∗∗∗^	−5.403 (0.001)^∗∗∗^
tpe.transitive	−0.024 (0.001)^∗∗∗^	1.330 (0.002)^∗∗∗^	
tpe.cyclic	−1.792 (0.003)^∗∗∗^		−1.760 (0.003)^∗∗∗^
tpe.twopath		−2.259 (0.004)^∗∗∗^	−0.040 (0.001)^∗∗∗^
AIC	10,075,300.988	10,075,300.988	10,075,300.988
Num. obs.	4,852,052	4,852,052	4,852,052

^∗∗∗^ *p*<0.001

## Visual validation and exploration

The previous section analyzed, for the three types of interaction events (dyadic undo, dyadic redo, and third-party edits), the consistency of the hierarchical interpretation with local dynamic patterns. In this section we want to explore the global hierarchy visually. As before we focus on assessing the difference between dominance derived from dyadic interaction and dominance derived from third-party edits. In the following we first define pairwise dominance orderings derived from the different types of interaction events, then we propose a simple way to assign a global hierarchy index to the editors of Wikipedia articles, and last but not least we derive and discuss network visualizations showing the distribution of hierarchy and highlighting dominance ties pointing in the wrong direction.

### Deriving pairwise dominance ordering

For two users *A* and *B* contributing to the same article we define a pairwise dominance order based on the empirical probabilities for undo, redo, and third party edits (that is, based on the values *r* *a* *t* *i* *o*.*u* *n* *d* *o*_*i*_(*A*,*B*), *r* *a* *t* *i* *o*.*r* *e* *d* *o*_*i*_(*A*,*B*), and *r* *a* *t* *i* *o*.*t* *p* *e*_*i*_(*A*,*B*)). For dominance derived from third-party edits, we have to define how to combine the values *r* *a* *t* *i* *o*.*t* *p* *e*_*i*_(*A*,*B*) and *r* *a* *t* *i* *o*.*t* *p* *e*_*i*_(*B*,*A*); for dominance derived from dyadic interaction we additionally have to define how to combine the values *r* *a* *t* *i* *o*.*u* *n* *d* *o*_*i*_(*A*,*B*) and *r* *a* *t* *i* *o*.*r* *e* *d* *o*_*i*_(*A*,*B*).

So, let *A* and *B* be two users contributing to a given Wikipedia article. We define the relative dominance order after revision *r*_*i*_ based on third-party edits among *A* and *B* by

$$order.tpe_{i}(A,B)=ratio.tpe_{i}(A,B)-ratio.tpe_{i}(B,A)\enspace. $$

**Fig. 4:**
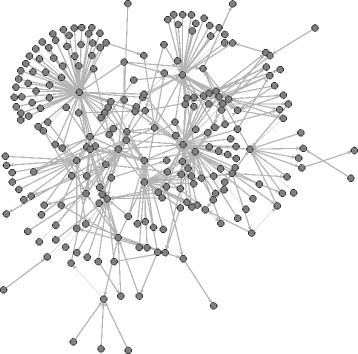
Pairwise dominance ordering based on third-party edits among contributing users of the Wikipedia article “The Third Man”. The layout has been determined with stress minimization and shows only the largest connected component of the network

Since *r* *a* *t* *i* *o*.*t* *p* *e*_*i*_(*A*,*B*) lies between zero and one, the difference *o* *r* *d* *e* *r*.*t* *p* *e*_*i*_(*A*,*B*) is in the interval [−1,1] and the values are skew-symmetric, i. e., it holds that *o* *r* *d* *e* *r*.*t* *p* *e*_*i*_(*A*,*B*)=−*o* *r* *d* *e* *r*.*t* *p* *e*_*i*_(*B*,*A*). For the network visualizations proposed later in this section we insert the edge (*A*,*B*) if and only if *o* *r* *d* *e* *r*.*t* *p* *e*_*i*_(*A*,*B*) is positive. Thus, two users *A* and *B* are connected by at most one (positively valued) edge, either (*A*,*B*) or (*B*,*A*); the value of the reverse edge is implied. Two users *A* and *B* are not connected by any edge if *o* *r* *d* *e* *r*.*t* *p* *e*_*i*_(*A*,*B*)=0, which happens for instance if they do not interact at all. In all images we show only the largest connected component of the network and all images show the state of the network at the end of our observation period (June 2016). Figure 4 shows these edges derived from the Wikipedia article “The Third Man.” This layout has been computed via stress minimization (Brandes and Pich 2008). In particular, the layout does not attempt to display a global hierarchy (see below for such images).

The pairwise dominance ordering based on dyadic undo and redo is derived in a similar way, however, we have to clarify how we combine the values *r* *a* *t* *i* *o*.*u* *n* *d* *o*_*i*_(*A*,*B*) and *r* *a* *t* *i* *o*.*r* *e* *d* *o*_*i*_(*A*,*B*). Results from the previous section indicated that the hypothesis that redo events go from lower to higher in the hierarchy is the most questionable. However, a redo event from *A* directed to *B* still attenuates a possible undo event on the same dyad. Indeed, if *A* sometimes makes edits of *B* undone and sometimes restores *B*’s edits, then this indicates a less negative evaluation than if *A* always erases but never restores *B*’s edits. Based on these considerations, we propose to define dyadic dominance via

$$dyadic.dom_{i}(A,B)=\max[\!0,ratio.undo_{i}(A,B)-ratio.redo(A,B)]\enspace. $$

Thus, the dyadic dominance from *A* to *B* is the difference between the undo and redo probability but it is at least zero. If *r* *a* *t* *i* *o*.*u* *n* *d* *o*_*i*_(*A*,*B*)<*r* *a* *t* *i* *o*.*r* *e* *d* *o*(*A*,*B*) then the value does not become negative (which would mean that *B* dominated *A*) but just indicates that *A* does not dominate *B*. Finally, the pairwise dominance order based on dyadic interaction is defined in the same way as for third-party dominance, namely by taking the difference between the dominance value on a dyad (*A*,*B*) and the value on the reverse dyad (*B*,*A*).

$$order.dyadic_{i}(A,B)=dyadic.dom_{i}(A,B)-dyadic.dom_{i}(B,A)\enspace. $$

**Fig. 5:**
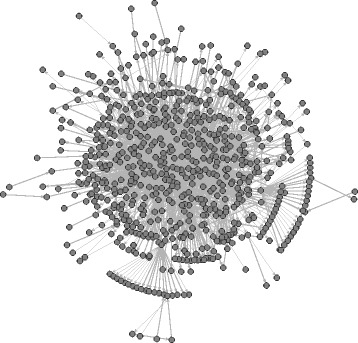
Pairwise dominance ordering based on dyadic dominance among editors of the Wikipedia article “The Third Man”. The layout has been determined with stress minimization and shows only the largest connected component of the network

As for third-party induced dominance order, the values *o* *r* *d* *e* *r*.*d* *y* *a* *d* *i* *c*_*i*_(*A*,*B*) lie also in the interval [−1,1] and are skew-symmetric. For the network visualizations we introduce a weighted edge from *A* to *B* if and only if *o* *r* *d* *e* *r*.*d* *y* *a* *d* *i* *c*_*i*_(*A*,*B*) is positive. In all images we show only the largest connected component of the network. Figure 5 shows these edges derived from the Wikipedia article “The Third Man.” This layout has been computed via stress minimization (Brandes and Pich 2008). In particular, it does not attempt to display a global hierarchy (see further below for such images).

### Deriving a hierarchy index

Based on the pairwise dominance orderings defined above we want to assign values to users expressing their hierarchical position as a function of their editing activity. We propose to take for such a measure for a user *A* the sum of all dominance values from *A* directed to any user minus the sum of all dominance values from any user directed to *A*. Concretely, the hierarchy index of user *A* based on third-party induced dominance is defined to be

$$hierarchy.tpe_{i}(A)=\sum\limits_{B\not=A}order.tpe_{i}(A,B)\enspace. $$

Note that the value *o* *r* *d* *e* *r*.*t* *p* *e*_*i*_(*A*,*B*) is positive if *A* dominates *B* and negative if *B* dominates *A*. Likewise the hierarchy index of user *A* based on dyadic interaction is defined to be

$$hierarchy.dyadic_{i}(A)=\sum\limits_{B\not=A}order.dyadic_{i}(A,B)\enspace. $$

Computing the position in a hierarchy via degree centrality is arguably one of the most primitive ways to do so—but also one of the most transparent and intuitively interpretable. We recall that our goal is not to come up with an optimal representation of global hierarchy, but rather to assess which of the two kinds of interaction events, dyadic interaction or third-party edits, can be more reliably interpreted as establishing or revealing a hierarchical order. Taking a simple and transparent way to derive a hierarchy makes the assessment of the relative appropriateness easier—even though more sophisticated ways to compute a global index from pairwise interaction could lead to a better ranking.

### Visual analysis of hierarchies

**Fig. 6:**
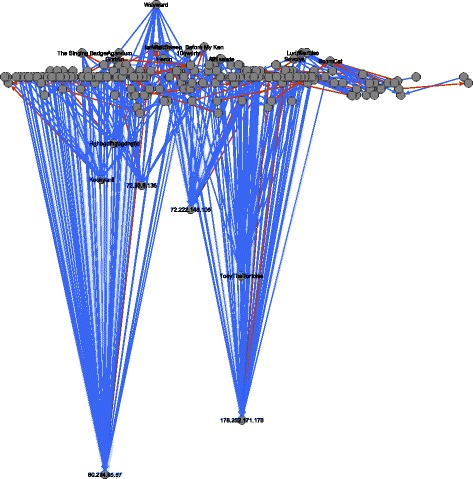
Hierarchy derived from third-party edits among editors of the Wikipedia article “The Third Man.” The *y*-coordinate of node *A* is proportional to the value *h* *i* *e* *r* *a* *r* *c* *h* *y*.*t* *p* *e*(*A*). Dominance edges pointing in the wrong direction (i. e., from lower to higher) are *colored red*

**Fig. 7:**
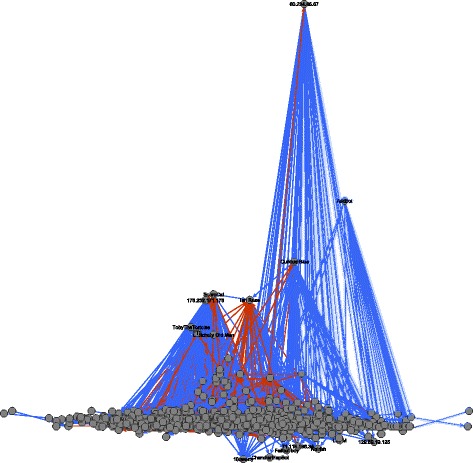
Hierarchy derived from dyadic interaction among editors of the Wikipedia article “The Third Man.” The *y*-coordinate of node *A* is proportional to the value *h* *i* *e* *r* *a* *r* *c* *h* *y*.*d* *y* *a* *d* *i* *c*(*A*). Dominance edges pointing in the wrong direction (i. e., from lower to higher) are *colored red*

**Fig. 8:**
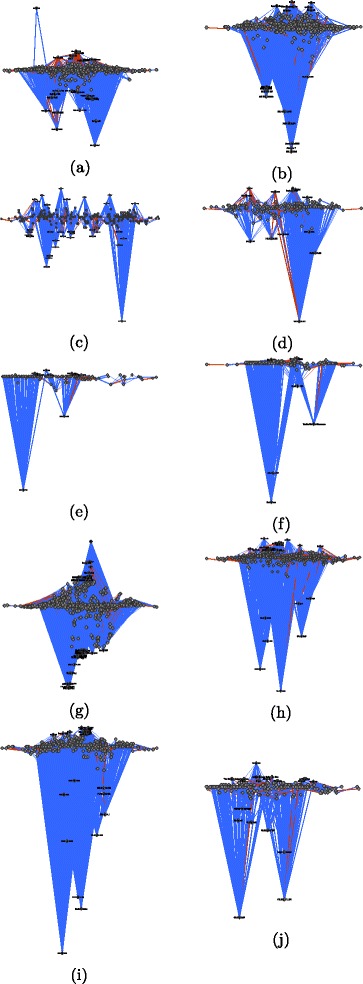
Hierarchy derived from third-party induced dominance among contributing users of various Wikipedia articles. The *y*-coordinate of node *A* is proportional to the value *h* *i* *e* *r* *a* *r* *c* *h* *y*.*t* *p* *e*(*A*). Dominance edges pointing in the wrong direction (i. e., from lower to higher) are *colored red*. **a** Balika Vadhu. **b** Ganymede (moon). **c** Greed. **d** Jay Park. **e** List of Hollyoaks locations. **f** Mothra. **g** Pea. **h** Shiv Sena. **i** Swimsuit. **j** The Third Man

**Fig. 9:**
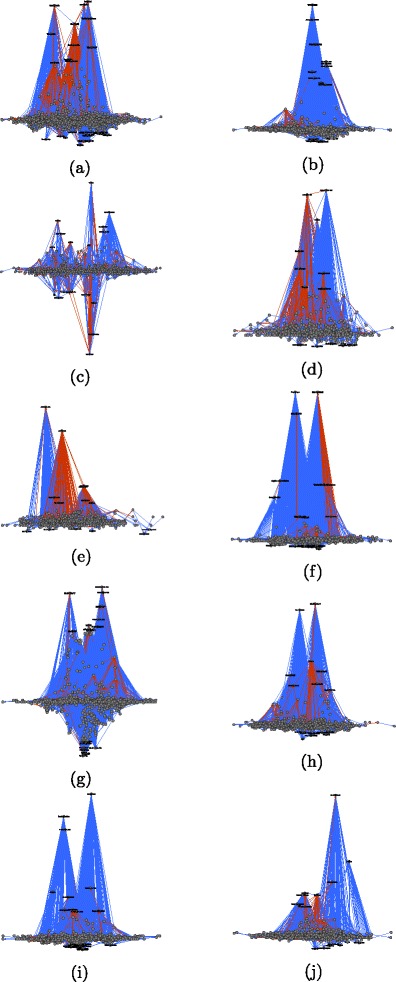
Hierarchy derived from dyadically induced dominance among editors of various Wikipedia articles. The *y*-coordinate of node *A* is proportional to the value *h* *i* *e* *r* *a* *r* *c* *h* *y*.*d* *y* *a* *d* *i* *c*(*A*). Dominance edges pointing in the wrong direction (i. e., from lower to higher) are *colored red*. **a** Balika Vadhu. **b** Ganymede (moon). **c** Greed. **d** Jay Park. **e** List of Hollyoaks locations. **f** Mothra. **g** Pea. **h** Shiv Sena. **i** Swimsuit. **j** The Third Man

To visualize the hierarchies as defined in the prior section, we use node-link diagrams where the *y*-coordinate of a node representing user *A* is proportional to *h* *i* *e* *r* *a* *r* *c* *h* *y*.*t* *p* *e*_*i*_(*A*) (or *h* *i* *e* *r* *a* *r* *c* *h* *y*.*d* *y* *a* *d* *i* *c*_*i*_(*A*), respectively). The *x*-coordinates are computed via constrained stress minimization (Brandes and Pich 2008) where the *y*-coordinate is fixed to the given values. In these images we color dominance edges in red if they point in the wrong direction, that is if they point from lower to higher hierarchical positions (edges that point in the right direction are colored blue). Usernames, respectively IP addresses of anonymous users, for the highest and lowest users are given as node labels. Figure 6 visualizes the hierarchy derived from third-party induced dominance for the Wikipedia article “The Third Man” and Fig. 7 visualized the respective hierarchy derived from dyadic interaction. The hierarchies derived from third-party edits for the ten sampled articles are displayed in Fig. 8 and those from dyadic interaction in Fig. 9.

It is striking that the anonymous user 60.234.65.67, which is the lowest in the hierarchy derived from third-party dominance, is the highest in the hierarchy derived from dyadic dominance. A simple inspection of the contributions of 60.234.65.67 reveals that this is clearly a vandal who once (precisely on 4 January 2006 at 08:15) deleted the whole text of the article “The Third Man” and replaced it by spam. This revision was reverted two minutes later by user Wayward. This example illustrates how dominance strictly defined on the basis of dyadic interaction may be misleading: a user can claim dominance for herself/himself by undoing other users’ edits. The situation is very different for third-party assigned dominance where third users assign a dominance order among two others. This altercentric view of dominance hierarchies (Podolny 2001) is consistent with the interpretation of dominance as conferred by others through status-conferring gestures (Gould 2002), and with recent work on the network dynamics of social status (Torlò VJ and Lomi 2017).

More generally we observe that in the hierarchy derived from third-party edits, the lowest users are mostly anonymous (labeled by their IP addresses) and the highest ones are mostly users who created an account. This distinction is more blurred in the hierarchy derived from dyadic interaction. (We can observe this pattern in the networks associated with all ten articles.) While an anonymous user is not necessarily a vandal and a registered user does not necessarily make high-quality edits, it is nevertheless plausible that being registered improves and facilitates recognition.

An additional general pattern revealed by graphical exploration is that a larger share of dominance edges point in the wrong direction, i.e., upward, in the hierarchies derived from dyadic interaction (displayed in red). Again, this pattern can be found in the networks associated with all of the ten articles that we analyzed (see Table 12).

**Table 12:** For the ten analyzed articles and the two ways to derive a hierarchy (third-party induced dominance and dyadically induced dominance): number of edges, number of reverse edges (i. e., those that point in the wrong direction, i. e., from lower to higher), and percentage of reverse edges

	Third-party dominance			Dyadic dominance		
Title	# edges	# reverse	% reverse	# edges	# reverse	% reverse
Balika Vadhu	2082	261	12.5%	6098	1801	29.5%
Ganymede (moon)	1954	123	6.3%	3228	709	22.0%
Greed	482	83	17.2%	1316	315	23.9%
Jay Park	406	60	14.8%	1676	596	35.6%
List of Hollyoaks …	179	27	15.1%	1976	660	33.4%
Mothra	363	25	6.9%	1490	269	18.1%
Pea	7566	363	4.8%	8566	769	9.0%
Shiv Sena	931	142	15.3%	2415	587	24.3%
Swimmsuit	905	78	8.6%	1891	366	19.4%
The Third Man	386	64	16.6%	2214	634	28.6%

Note that for all articles, the hierarchy derived from dyadic interaction has a higher percentage of edges pointing in the wrong direction. Note that all numbers are for the largest connected component of the respective network

In summary, the visual exploration of hierarchies tends to offer qualitative support for the results produced by statistical analysis: evidence of dominance order derived from third-party edits is more robust than that derived from dyadic interaction. This conclusion suggests that dyadic and triadic structures of interaction must be considered jointly in the analysis of hierarchical order in Wikipedia’s production system.

## Conclusions

We proposed and implemented new methods to derive and analyze three types of interaction events from co-editing Wikipedia articles: dyadic undo, dyadic redo, and third-party edits. A combination of dyadic undo with dyadic redo gives rise to dyadic dominance where a user *A* claims to dominate another user *B*. Third-party induced dominance, in contrast, arises when a third user *C* restores edits of user *A* that have been undone by user *B*.

Building on core theoretical concepts in behavioral ecology, the main objective of our paper was to examine the extent to which dyadic and triadic interactions are consistent with a linear hierarchical ordering in the Wikipedia editing network. We focused on antisymmetry and transitivity in temporal sequences of editing events as the main *constructive mechanisms* underlying the formation of linear dominance hierarchies. We specified and estimated new relational event models for signed multiplex networks to search for traces of hierarchical ordering in the production of Wikipedia articles.

The analysis revealed that past editing events have two distinct effects on future interaction among Wikipedians: on the frequency of events in the undirected dyad {*A*,*B*}, dyad and on the relative dominance of the directed dyad (*A*,*B*) over (*B*,*A*). The effects on the undirected dyads are often more consistent with structural balance theory according to which undo events and third-party edits may be interpreted as revealing negative ties. The effects on the directed dyads are often more consistent with a hierarchical interpretation. This finding is similar to that reported in Leskovec et al. (2010) in a study of voting behavior among Wikipedians. We also showed that the effect on the event frequency may obfuscate effects on the hierarchical ordering. This finding is similar to that reported in Lerner (2016) where effects on the interaction frequency were separated from effects influencing the sign of ties. The analysis in our paper also revealed that the three different types of events show different levels of consistency with the linear hierarchical ordering hypothesis. Most notably, third-party edits are the only type of event that is anti-reciprocal, irrespective of whether we control for a change in the interaction frequency. When third-party interventions are ignored, dyadic undo events are inconsistent with linear hierarchical ordering.

The visual exploration of hierarchies revealed the frailty of strict dyadic dominance as a basis of hierarchical order: individual users can just claim to dominate others by undoing their edits. In contrast, third-party assigned dominance requires a third user (which, following conventions adopted in behavioral ecology, we called “bystander”) to adjudicate dominance. Hierarchies derived from third-party edits typically had anonymous users occupying lowest positions in the hierarchy, and registered users in the highest positions, confirming the intuition that registering is necessary for acquiring credibility and status. More generally, third-party dominance implied a smaller share of edges inconsistent with a linear hierarchy than dyadic dominance.

The narrow focus of the paper on the consistency of local interaction conditions with a global hierarchal ordering, blinded us to alternative interpretations that remain contextually possible – even if not directly relevant to the analytical purposes of the paper. At least two such interpretations deserve mention as they may provide an alternative framing for *interpreting* the results of our study. More specifically, edit events might be also affected by the (i) quality of the deleted or restored text (the *quality interpretation*), and (ii) political or ideological orientations of the editors (the *polarization interpretation*). These alternative interpretations occasionally, but not necessarily, lead to predictions that are different from those suggested by the theory of dominance hierarchy that we have proposed.

The *quality interpretation* would explain edit events as determined uniquely, or mainly by opinions that editors hold about quality of the deleted or restored text. Thus, an undo event from user *A* directed to user *B* would indicate that *A*’s quality requirements for contributions to Wikipedia articles are higher than *B*’s. The quality interpretation would lead to exactly the same hypotheses for the configurations in Fig. 1 as the hierarchical interpretation and would support the same predictions. We note, however, that the *quality interpretation* is, in fact, an *interpretation* as text edit events are directly observable, while the quality of text being edited is not. Quality of text is difficult to measure uniquely or unambiguously. It is also unclear that considerations of quality may be generally applicable to sub-sets of words within an article – rather than to the article as a whole. For example, the internal Wikipedia quality rating system is defined at the level of articles, rather than single chunks of text within the article. We also note that the *quality interpretation* implies that the quality of Wikipedia articles would increase monotonically with the number of edits – because edits would be driven exclusively by quality considerations. Testing this hypothesis is possible but beyond the objectives of our study. These considerations notwithstanding, there is no doubt that, if measured correctly, the quality of text could be an important contextual factor affecting sequences of text edits.

The *polarization interpretation* would explain edit events as revealing membership of users in opposing opinion factions. In this interpretation, an undo event from user *A* directed to user *B* would indicate that *A* and *B* militate in factions with opposing political orientations. Interestingly, the *polarization interpretation* would give rise to hypotheses that differ from those sustained by the hierarchical ordering (and the quality) interpretation. In particular, the *polarization interpretation* would support different predictions for reciprocation and for transitive closure. Indeed, if user *B* initiated an undo event targeted at user *A* in the past, then this would indicate that *A* and *B* are in different factions and, therefore, a future undo event from *A* to *B* is *more* likely than on a random pair of users. (Instead, both the hierarchical and the quality interpretation would predict a decreased undo probability from *A* to *B*.) Turning to transitive closure, if it happened in the past that *A* had an undo event targeted at some other user and this other user had an undo event targeted at *B*, then *A* would be in a different camp than this other user which, in turn is in a different camp than *B*. Thus, assuming the presence of only two opinion factions, *A* and *B* are in the same faction and thus an undo event from *A* to *B* is *less* likely than on a random dyad (Instead, both the hierarchical and the quality interpretation would predict an increased undo probability from *A* to *B*). We note that in the presence of more than just two opinion factions, the polarization interpretation would sustain no specific predictions for transitive closure. The polarization interpretation makes the same predictions for inertia and cyclic closure as the hierarchical (and, thus, the quality) interpretation, but it does not support unique predictions about the various degree effects. We note that the polarization interpretation is consistent with structural balance theory (Heider 1946; Cartwright and Harary 1956) when interpreting undo events as revealing negative ties.

While the possibility of alternative interpretations of our result are clearly deserving of further consideration, we think that the interpretation we offer has the unique advantage of hinging on a general theoretical framework for predicting editing events – and not just for interpreting empirical regularities in sequences of editing events. Interestingly, we also note that, in principle, the empirical value of alternative interpretations may be tested directly by adopting the analytical approach and the statistical methods that we have proposed and implemented in this paper.

Quality consideration and the possibility of polarization and contention organized into non-overlapping factions of users are clearly important contextual factors that may inject powerful and realistic non-linear elements in the hierarchical order of the Wikipedia edit network. Despite this qualification, we think that the results we have reported, however preliminary, provide a new bridge between micro-foundational theories of social behavior, and the global organizational structure of one of the largest, most popular, and most successful open production projects currently in existence.

## Endnotes

^1^ www.wikipedia.org

^2^ Here we resolve 0/0 to be equal to 0, since no event of that type could have happened so far on such a dyad.

^3^ These turned out to be the articles selected in our sample: Balika Vadhu; Ganymede (moon); Greed; Jay Park; List of Hollyoaks locations; Mothra; Pea; Shiv Sena; Swimsuit; and The Third Man.
